# Miscellaneous Medical Intelligence

**Published:** 1852-03

**Authors:** 


### ARTICLE X.

### MISCELLANEOUS MEDICAL INTELLIGENCE.

We would call the attention of our friends to the Card of the Sec'y of the Amer. Med. Assoc'n in another department of the Journal. Let all of our Societies and Institutions entitled,to it, be represented. It will furnish a good opportunity to visit Washington City while Congress is in session to those curious in such matters, as well as to keep up the representation of the North-West, in our National Medical Congress.

A Physician in the interior of Indiana wishes us to announce that he has discoverd a specific for Milk Sickness. We can only comply, upon his furnishing us with a full account of his mode of treatment.

It is rumored that the venrable Prof. Mott, is to return to the Universiry of the City of New York, and that Dr. Van Buren is to fill the chair of Anatomy made vacant by the death of Prof. Pattison.

Dr. A. F. Stevens of Hanover, Ills., reports in a private letter a case of twins, with distinct membranes and placentæ, in which both children were born by the breech presentation. The boy weighed 9½ lbs., and the girl 8 lbs., making an aggregate of 17½ lbs.

It is reported that Dr. John Bell has resigned the Chair of Practice in the Medical College of Ohio, and will return to Philadelphia.

Profs. Wright and Skinner have resigned their Chairs in the new Medical School in Cincinnati organized last winter,called the Cincinnati College of Medicine and Surgery. Applications for the chair of Chemistry are solicited by Prof A. H. Baker, Cincinnati, O., on behalf of the Trustees.

The Faculty of the Indiana Central Medical College at Indianapolis, has been re-organized. As it now stands, Profs. Deming, Downey and Bobbs retain the Chairs they formerly held. R. J. Patterson, M. D., has that of Anatomy, C. G. Comeygs, M. D. Materia Medica, and L. E. Leonard, M. D. Obstetrics, &c.

Prof. Dalton's course of Lectures in the Buffalo University, has given the greatest satisfaction to all concerned.

It is stated that the number of Physicians in Paris has been greatly reduced within a few years. What the position of Medical matters there will be, under the usurpation, we have not been able to learn.

Several of our cotemporaries are publishing articles upon Spirit Rapping's, Influences, and the like. Since witch-craft has gone out of date in its old form by neglect, we think that it will in its new phase if left alone.

